# Moth-eye Structured Polydimethylsiloxane Films for High-Efficiency Perovskite Solar Cells

**DOI:** 10.1007/s40820-019-0284-y

**Published:** 2019-06-25

**Authors:** Min-cheol Kim, Segeun Jang, Jiwoo Choi, Seong Min Kang, Mansoo Choi

**Affiliations:** 1 Global Frontier Center for Multiscale Energy Systems, Seoul National University, Seoul, 151-744 Republic of Korea; 2 Department of Mechanical and Aerospace Engineering, Seoul National University, Seoul, 151-744 Republic of Korea; 3 Department of Mechanical Engineering, Hanbat National University, Daejeon, 34158 Republic of Korea; 4 Department of Mechanical Engineering, Chungnam National University, Daejeon, 34134 Republic of Korea

**Keywords:** Polydimethylsiloxane films, Moth-eye, Photolithography, Perovskite solar cells, Photovoltaic

## Abstract

Large-area polydimethylsiloxane (PDMS) films with variably sized moth-eye structures were fabricated to improve the efficiency of perovskite solar cells. An approach that incorporated photolithography, bilayer PDMS deposition and replication was used in the fabrication process. By simply attaching the moth-eye PDMS films to the transparent substrates of perovskite solar cells, the optical properties of the devices could be tuned by changing the size of the moth-eye structures. The device with 300-nm moth-eye PDMS films greatly enhanced power conversion efficiency of ~ 21% due to the antireflective effect of the moth-eye structure. Furthermore, beautiful coloration was observed on the 1000-nm moth-eye PDMS films through optical interference caused by the diffraction grating effect. Our results imply that moth-eye PDMS films can greatly enhance the efficiency of perovskite solar cells and building-integrated photovoltaics.

**Electronic supplementary material:**

The online version of this article (10.1007/s40820-019-0284-y) contains supplementary material, which is available to authorized users.

## Highlights

- Moth-eye structured polydimethylsiloxane (PDMS) films with different sizes were fabricated to improve the efficiency of perovskite solar cells.
- The PDMS with 300-nm moth-eye films significantly reduced light reflection at the front of the glass and therefore enhanced the solar cell efficiency of ~ 21%.
- The PDMS with 1000-nm moth-eye films exhibited beautiful coloration.

**Electronic supplementary material:**

The online version of this article (10.1007/s40820-019-0284-y) contains supplementary material, which is available to authorized users.

## Graphical abstract

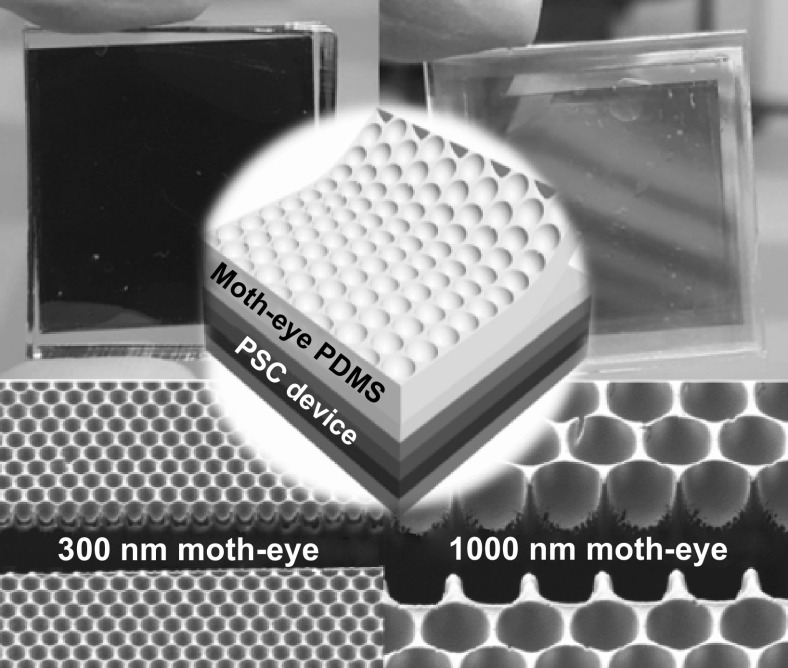

## Introduction

Since a renewable energy device with a power conversion efficiency (PCE) of 3.8% appeared firstly in 2009, organic/inorganic perovskite solar cells (PSCs) have received a great deal of attention as solar devices due to their super photovoltaic properties [1–6]. Advanced efforts to construct highly efficient PSCs in recent years have led to PCEs exceeding 20% with good reproducibility [7–9]. These high-efficiency devices exhibit an average photocurrent density of ~ 24 mA cm^−2^ with similarly high external quantum efficiency (EQE) values along the entire wavelength [7]. Although this is lower than the theoretical maximum photocurrent density of ~ 26 mA cm^−2^, it has been noted that the best strategy for improving the PCE of photovoltaic devices is to enhance photocurrent density by increasing the absolute value of EQE [10]. Therefore, the efficiency of exterior sunlight absorbance is critical for evaluating the performances of photovoltaic devices, even when the same materials and methods are used for their fabrication [11]. Doped substrates, such as F-doped SnO_2_ (FTO), indium tin oxide (ITO), and graphene, are typically used to make conductive electrodes for PSCs. However, compared to bare substrates, doped substrates reduce the transmission of incident light [12]. Thus, improving light-harvesting efficiency (LHE) by optical modulation is important for maximizing the efficiency of PSC devices.

To increase the LHE of solar energy systems, antireflective surfaces [10, 13–15], light-scattering layers [16–18], and plasmonic photonic crystals [19–21] have been developed and applied. Biomimetic soft lithography is a promising alternative for increasing the PCE of PSCs; it involves simply coating or attaching a material to the external surface of a transparent substrate. Antireflective nanostructures inspired by the eye of a moth demonstrate superior structural antireflectivity over a wide range of wavelengths. These uniformly arranged nanostructures induce a gradual refractive index gradient at the surface [11, 22]. In terms of materials, polydimethylsiloxane (PDMS) is frequently used for fabricating bioinspired structures using soft lithography. In previous work, we showed that the properties of PDMS can be applied effectively in photovoltaic devices [14]. Recent efforts to increase the PCE of PSCs have employed biomimetic multiscale architecture approaches [23–26]. However, these approaches were applied to conventional devices with irregular microstructures obtained through expensive and complex fabrication processes. They did not employ uniformly arranged moth-eye nanostructures. The diffraction grating effect at visible wavelengths from 300 to 800 nm was therefore not considered, which made it difficult to exceed a PCE of 20%. Novel criteria and a standard methodology are thus required to fabricate well-ordered bioinspired optical structures that can be introduced to highly efficient PSCs.

In this paper, we report an optimized PDMS nanostructure polymer film with inverted moth-eye features for effective utilization of efficient PSCs. Using a PDMS soft lithography method, we successfully fabricated well-ordered, sharp, inverted moth-eye nanostructures with high fidelity. The fabricated bioinspired polymeric surface demonstrated superior optical and antireflective properties as well as beautiful coloration from the diffraction grating effect. We compared 300-nm and 1000-nm periodic nanostructures to identify a critical dimension for enhancing the EQE of the photovoltaic devices based on the diffraction grating equation and comprehensive experiments. By simply attaching the bioinspired film to the transparent substrate of a PSC via Van der Waals forces, the optical properties were improved considerably over those of a reference device. Finally, the photocurrent density of the devices with 300-nm periodic grating structures was improved by 5.4% over that of the reference due to enhanced LHE. Consequently, PCE in the PSCs reached up to ~ 21%. Furthermore, colorful photovoltaic devices were obtained using the 1000-nm grating structures, which could be adapted for various applications, particularly in building-integrated photovoltaics (BIPV).

## Experimental Section

### Preparation of Moth-eye Silicon Masters

The detailed fabrication process has been described previously [11]. To summarize it briefly here, a 1000-nm-thick photoresist (LX-429, Dongjin Semichem, Korea) film was spin-coated onto a clean 8-inch silicon wafer. The coated photoresist was exposed to a KrF laser source with hexagonal array masks with pattern diameters of 170 or 600 nm. Each patterned silicon wafer was anisotropically etched with an inductively coupled plasma (ICP) system to obtain pillar structures with depths of 180 or 500 nm, respectively. After removing the photoresist layer, a 100-nm or 330-nm-thick SiO_2_ layer was deposited on the pillared wafer surface by thermal oxidation under flowing H_2_ and O_2_ gas. Finally, hexagonally close-packed 300-nm and 1000-nm moth-eye arrays (diameter, height, and period were equal to 300 and 1000 nm, respectively) were completed following deposition of a 10-nm-thick nitride layer.

### Fabrication of Moth-eye PDMS Films

To reduce the surface energy of each moth-eye master, gaseous deposition of a very thin C_4_F_8_ layer was performed by an ICP system supplied with C_4_F_8_ gas at approximately 100 standard L min^−1^ at 22 mTorr for 1 min. To replicate the prepared structures with high pattern fidelity, two different PDMS materials (hard-PDMS (h-PDMS) and soft-PDMS (s-PDMS)) were used. A highly viscous h-PDMS solution was prepared by mixing 1.7 g VDT-731 vinyl PDMS pre-polymer (Gelest Corp., Germany), 0.5 g HMS-301 hydrosilane pre-polymer (Gelest Corp., Germany), 10 μL SIP6831.2 platinum catalyst (Gelest Corp., Germany), and 5 μL 2,4,6,8-tetramethyltetravinylcyclotetrasiloxane modulator (Sigma-Aldrich, USA) with magnetic stirring at 2000 rpm for 10 min. The h-PDMS solution was poured onto the moth-eye silicon master and coated in an approximately 20-µm-thick layer by doctor-blading deposition. The h-PDMS layer was cured in an oven at 80 °C for 20 min. A 1:10 solution of s-PDMS (base) and curing agent was made with a Sylgard^®^ 184 kit (Dow Corning, USA), cast onto the h-PDMS supporting layer, and cured in an oven at 80 °C for 1 h. Finally, the replicated 300-nm and 1000-nm inverted moth-eye PDMS (h-PDMS/s-PDMS) films were detached from the silicon masters.

### Fabrication of Perovskite Solar Cells

All chemical solutions for PSC fabrication were purchased from Sigma-Aldrich (USA) and used as received. Poly(bis(4-phenyl)-(2,4,6-trimethylphenyl)amine) (PTAA) was purchased from Xi’an Co. (China). Lead iodide was purchased from Alfa Aesar. Methylammonium bromide (MABr), methylammonium chloride (MACl), and formamidinium iodide (FAI) were purchased from Dyesol (Australia). The glass/ITO substrate was cleaned sequentially with acetone, isopropanol, and distilled water in an ultrasonicator. The substrate was spin-coated with PTAA in chlorobenzene solution (2 mg mL^−1^) at 6500 rpm for 30 s and annealed at 100 °C for 10 min. A 1.3 M PbI_2_ solution in 9.5:0.5 DMF/DMSO and a 1 mL solution of FAI (60 mg), MABr (6 mg), and MACl (6 mg) in IPA were prepared for fabrication of the perovskite films. The PbI_2_ solution was spin-coated onto the PTAA thin layer at 2500 rpm for 30 s, and then, the mixed organic halide solution was distributed on the semi-transparent PbI_2_ film by dripping. The perovskite film was spin-coated at 5000 rpm for 30 s and then annealed at 150 °C for 10 min. All spin-coating was performed in a dry room at a relative humidity of < 10% at 25 °C. For electron transport, C_60_ (20 nm) and BCP (10 nm) layers were deposited onto the perovskite layer by organic vacuum thermal evaporation at a rate of 0.2 Å s^−1^. A layer of Cu metal (50 nm) was then deposited on top of the BCP layer at 0.5 Å s^−1^ over a metal shadow mask to form the metal electrode. Each evaporation process was performed under a strong vacuum at 10^−7^ torr. Finally, we simply attached the moth-eye PDMS films onto the glass side of the PSCs.

### Characterization

Measurements for the *J*–*V* curves were performed at a scan rate of 0.4 mV ms^−1^ with a Keithley 2400 source meter (Tektronix, Beaverton, OR). An Oriel S013 ATM solar simulator (Newport Corp., Irvine, CA) was used for AM 1.5 G illumination at an intensity of 100 mW cm^−2^, followed by calibration with a 91150 KG5 filtered standard silicon reference solar cell. Measurements were carried out at 25 °C in a N_2_-filled glove box. Quantum efficiency was evaluated by incident photon-to-charge carrier efficiency (IPCE) analysis with an IQE-200 system (Newport, Beaverton, OR) equipped with a 100 mW Xe lamp and a lock-in amplifier. The transmittance and reflectance spectra were collected on a Cary 5000 UV–visible spectrometer (Agilent technologies, Santa Clara, CA). Atomic force microscopy (AFM) images were obtained with an NX10 AFM (Park Systems, Suwon, Korea) in contact mode using a NSC36/Cr–Au tip. Scanning electron microscopy (SEM) images were obtained with a Merlin field emission SEM (Zeiss, Oberkochen, Germany) equipped with an Auriga series focused ion beam (FIB).

## Results and Discussion

### Structure of Perovskite Solar Cells with Moth-eye PDMS Films

**Fig. 1:**
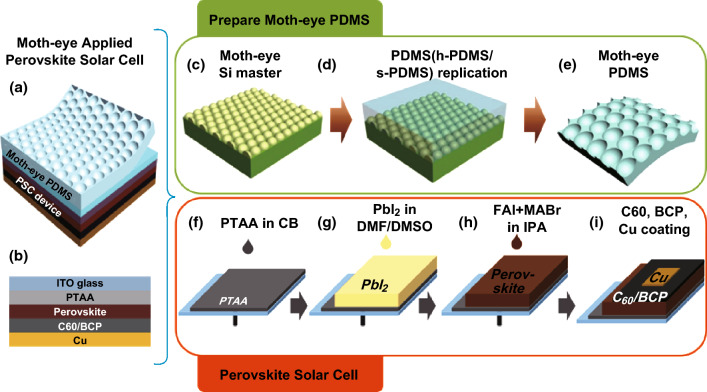
Representative schematic images of **a** completed PSC with moth-eye layer and **b** PSC configuration. **c** Fabrication of the hexagonally packed moth-eye array on a silicon wafer. **d** Replication of the master structure by successive coating and thermal curing of h-PDMS and s-PDMS. **e** Completed moth-eye PDMS film. **f** Spin-coating deposition of PTAA hole transport layer on ITO/glass substrate. **g** Spin-coating deposition of PbI_2_ thin film. **h** Fabrication of perovskite layer by additional spin-coating of FAI/MABr/MACl solution in IPA. **i** Deposition of C_60_/BCP electron transporting layer on Cu metal electrode

A representative schematic of a PSC with a moth-eye PDMS film is shown in Fig. 1a. The PSC was comprised of a glass/ITO electrode, a poly(bis(4-phenyl)-(2,4,6-trimethylphenyl)amine) (PTAA) hole transport layer, a perovskite active layer for photon absorption, a bathocuproine (BCP) buffer layer, a C_60_ electron transport layer, and a Cu electrode. The inverted moth-eye PDMS (h-PDMS/s-PDMS) film was gently affixed to the front side of the PSC by Van der Waals interactions between the rubbery s-PDMS surface and the ITO glass. The PSC schematic shown in Fig. 1b is based on a recent study on a state-of-the-art high-efficiency PSC [27].

The fabrication procedure for the moth-eye PDMS film is illustrated in Fig. 1c–e. First, a moth-eye silicon master with a well-defined hexagonal array of 300-nm or 1000-nm nanostructures was prepared by conventional photolithography and an anisotropic etching process. This was followed by successive deposition of SiO_2_ and nitride to form a compact, parabolic structure (Fig. 1c). Before replicating the structure, a thin C_4_F_8_ layer was deposited to reduce the surface energy of the moth-eye silicon masters, which was critical for successful demolding of the replicated PDMS films. The bilayer feature of the PDMS (h-PDMS/s-PDMS) film (Fig. 1d) was important for high-fidelity replication of the nanostructures and to ensure conformal contact with the glass surface. First, the prepared masters were coated with an approximately 20-µm-thick layer of h-PDMS with a high elastic modulus (~ 9 MPa) [28] by doctor-blading deposition. Pouring and curing of a ≤ 3-mm-thick s-PDMS layer with an elastic modulus of ~ 2 MPa [28] was performed on the supporting h-PDMS layer. A schematic of the complete replicated PDMS (h-PDMS/s-PDMS) with an inverted moth-eye structure is shown in Fig. 1e.

The inverted PSC in combination with the moth-eye PDMS is based on the PTAA hole transport layer. PTAA-based PSCs exhibit not only extremely high efficiency but also great long-term stability [29]. PTAA dissolved in chlorobenzene was spin-coated onto the ITO/glass to form the hole transport layer, as illustrated in Fig. 1f. The PbI_2_ thin film and the mixed FAI/MABr/MACl solution were then sequentially spin-coated onto the PTAA layer (Fig. 1g–h). The perovskite light-absorbing layer was composed of (FAPbI_3_)_0.97_(MAPbBr_3_)_0.03_ [27]. Finally, the C_60_/BCP electron transport layer and Cu metal electrode were added by evaporation deposition (Fig. 1i).

### Morphology of Completed Moth-eye PDMS Films and Perovskite Solar Cells

**Fig. 2:**
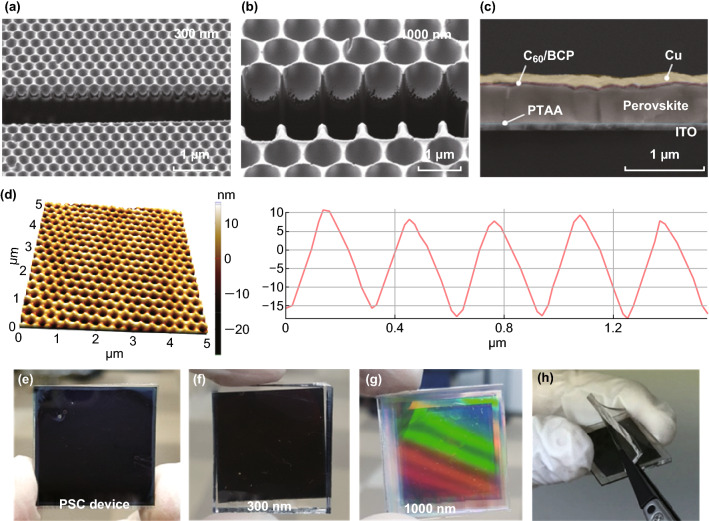
FIB-assisted cross-sectional SEM images of **a** 300-nm moth-eye PDMS film, **b** 1000-nm moth-eye PDMS film, and **c** PSC configuration imaged in BSE mode. **d** AFM image and line profile of 300-nm moth-eye PDMS film. Digital camera images of **e** completed PSC device, **f** PSC with 300-nm moth-eye PDMS film, **g** PSC with 1000-nm moth-eye PDMS film, and **h** detachment of 300-nm moth-eye PDMS film from PSC

FIB-assisted cross-sectional SEM images of the inverted 300-nm and 1000-nm moth-eye PDMS films are shown in Fig. 2a and 2b, respectively, and a completed PSC can be seen in Fig. 2c. Both the inverted 300-nm and 1000-nm moth-eye structures were successfully replicated from the silicon masters thanks to the C_4_F_8_ surface passivation process and employment of h-PDMS with a high elastic modulus. The back-scattered electron (BSE) image of the cross-sectional PSC configuration (Fig. 2c) showed that each of the ITO, PTAA, perovskite, C_60_/BCP, and Cu layers were well formed without any defects. To further investigate the sub-micron morphological features of the inverted 300-nm moth-eye PDMS films, AFM imaging was conducted in contact mode with constant force (Fig. 2d). Because the rubber-like PDMS surface was easily deformed by the AFM tip, accurate height measurement was not possible. However, the well-defined morphology of the moth-eye structure was clearly visible in the AFM images. Importantly, AFM analysis confirmed that the lateral pitch of the structure was approximately 300 nm. A digital camera image of a completed 2.5 × 2.5 cm^2^ PSC device is shown in Fig. 2e. Compared to the reference PSC device, the PSC with a 300-nm moth-eye PDMS film had dark coloration due to the antireflective properties of the moth-eye structure, which led to more light absorption in the perovskite layer. Interestingly, the PSC with a 1000-nm moth-eye PDMS film exhibited optical interference coloration due to external reflection from the structures. This phenomenon acted as diffraction gratings on the front of the PSC device in the visible wavelength range. It was difficult to obtain beautiful coloration on the PSC because the high absorption coefficient (α) of perovskite resulted in dark coloration. This optical characteristic can be adapted in diverse environments, where additional coloration of PSC is required, such as in BIPV. Furthermore, the moth-eye PDMS films were highly flexible due to the low elastic modulus of s-PDMS. Hence, it was relatively easy to attain conformal contact to the glass and to detach the moth-eye layer from it (Fig. 2h).

### Optical Properties of Moth-eye PDMS Films

To elucidate the optical characteristics of the moth-eye PDMS films, total transmittance spectra were collected from 300 to 800 nm with a UV–Vis-NIR spectrophotometer. The 300-nm moth-eye PDMS films on glass exhibited greatly enhanced optical transmittance over a broad wavelength range compared to the reference glass substrate; however, the 1000-nm moth-eye PDMS films on glass had reduced optical transmittance over the entire measured region compared to the reference. These phenomena can be explained by the well-known grating equation under normal incidence lighting (Eq. 1) [30].

1 $$\sin \theta_{\text{d}} = m\lambda /np$$

where *n* is the refractive index of the incident medium, *p* is the grating period, *m* is the order of the diffracted light, *λ* is the incident wavelength, and *θ*_d_ is the diffraction angle. The incident medium was air, where *n*_air_ ≈ 1. Based on Eq. 1, the 300-nm moth-eye PDMS film effectively reduced external reflection because its 300-nm periodicity suppressed external reflection at wavelengths between 300 and 800 nm. It therefore displayed greatly enhanced transmittance of up to approximately 96%, whereas the transmittance of the reference glass was approximately 92%. This result explains the darker coloration of the PSC with the 300-nm moth-eye film shown in Fig. 2f. In the case of the 1000-nm moth-eye film, reduced transmittance over the 300–800 nm range was a consequence of higher-order diffraction. In contrast to the 300-nm moth-eye structure, which exhibited no higher-order external reflection, the 1000-nm moth-eye displayed first-, second-, and even third-order external reflection due to the diffraction grating effect. This was confirmed by the iridescent coloration visible in the digital camera image in Fig. 2g.

**Fig. 3:**
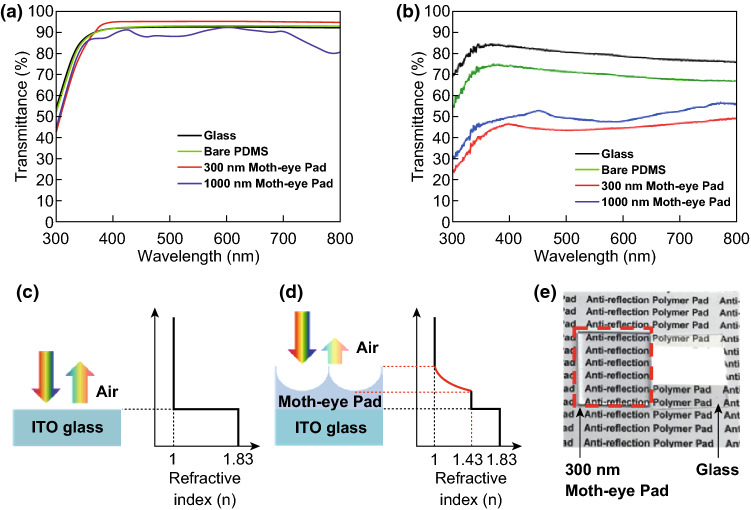
**a** Transmittance and **b** reflectance spectra of the prepared samples. Schematic refractive index profiles of the **c** ITO/glass substrate and **d** moth-eye PDMS film/ITO/glass substrate. **e** Digital camera image demonstrating the antireflective effect of the 300-nm moth-eye PDMS film

Further characterization of the optical properties of the 300-nm and 1000-nm moth-eye structures was carried out by reflectance spectral analysis. The reflectance spectra of the 300-nm moth-eye structure (Fig. 3b) showed an average reduced reflectance of approximately 4.3% compared to the reference value of approximately 7.9%, which is consistent with the enhanced transmittance seen in Fig. 3a. The 1000-nm moth-eye structure yielded greater reflectance than the 300-nm moth-eye structure. However, its reflectance was lower than that of the reference glass, which is not consistent with the transmittance results. We concluded that because of the higher-order diffraction in the 1000-nm moth-eye PDMS film (n ≈ 1.43), which occurred up to the fourth order, normally incident light was trapped in the film due to total internal reflection. This resulted in optical loss, which was calculated by Eq. 2 [31].

2 $$100 \, \left( \% \right) \, {-}{\text{ transmittance }}\left( \% \right) \, {-}{\text{ reflectance }}\left( \% \right)$$

Schematic illustrations of the antireflective effects of the 300-nm moth-eye PDMS film are shown in Fig. 3c, d. The 300-nm moth-eye structure did not exhibit higher-order external reflection; furthermore, its parabolic shape gradually changed the refractive index at the interface between air and the PDMS surface. Therefore, it effectively reduced interfacial Fresnel reflection. The antireflective effect of the 300-nm moth-eye PDMS film was confirmed by comparison with bare glass, as shown in Fig. 3e. With the 300-nm moth-eye PDMS film, the characteristics under the glass were seen clearly due to the effectively reduced external reflection at the surface. However, with bare glass, the characteristics were not easily discernible.

### Photovoltaic Performance of PSCs with Moth-eye PDMS Films

**Fig. 4:**
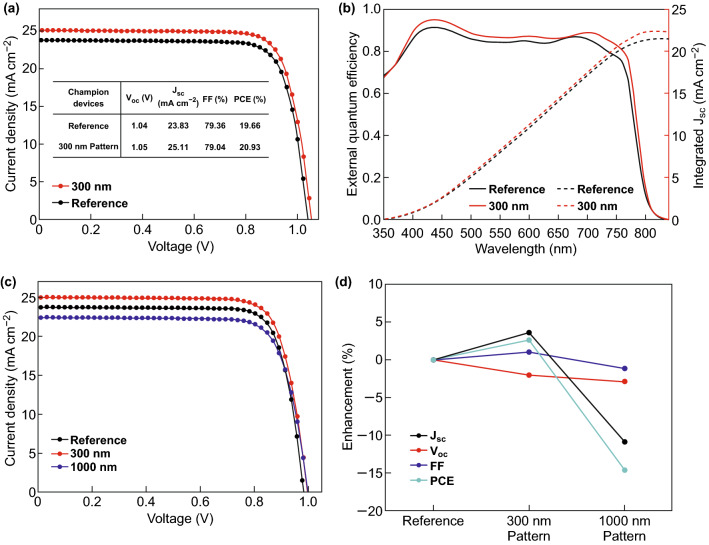
**a** Measured *J*–*V* curves and **b** external quantum efficiency spectra for the perovskite photovoltaic devices with the highest performance. Photovoltaic parameters of the reference device and the PSC made with a 300-nm moth-eye PDMS film are shown in the inset of **a**. **c** *J*–*V* curves for reference perovskite photovoltaic and devices made with 300-nm and 1000-nm moth-eye PDMS films. **d** Measured enhancement of photovoltaic parameters in PSCs made with 300-nm and 1000-nm moth-eye PDMS films relative to the reference device

Optical enhancements brought about by moth-eye PDMS films with nanostructures of different sizes were directly reflected in the photovoltaic performance of the PSCs. In particular, the current density (*J*_SC_) in the PSCs was considerably altered by both the 300-nm and 1000-nm moth-eye PDMS films. The device with the best performance was made with 300-nm moth-eye PDMS. Its power conversion efficiency (PCE) was 20.93%, and its *J*_SC_ was as high as 25.11 mA cm^−2^. The PCE of the reference device was only 19.66%, and its *J*_SC_ was 23.83 mA cm^−2^ (Fig. 4a). By addition of the 300-nm moth-eye PDMS, PCE was improved by 6.5%, and *J*_SC_ was improved by 5.4%. The increase in PCE is clearly attributable to enhanced light absorption due to the antireflective property of the 300-nm moth-eye PDMS, which was also evidenced by the IPCE measurement (Fig. 4b**)**. The integrated *J*_SC_ from the IPCE measurements was slightly lower (approximately 10%) than the *J*_SC_ from the *J*–*V* measurements. This is reasonable because IPCE measurements are conducted with monochromatic light, which has a lower intensity than one-sun solar irradiation [32, 33]. The device made with the 300-nm moth-eye PDMS had higher external quantum efficiency than the reference device over the entire wavelength range from 350 to 800 nm. While the 300-nm moth-eye PDMS improved PSC performance significantly, the purpose of the 1000-nm moth-eye PDMS was to display beautiful coloration when attached to a dark black PSC. The PCE in this device reached as high as 17.43%, and a *J*_SC_ of 22.43 mA cm^−2^ was observed, despite the optical interference from its larger-scale moth-eye pattern. Though J_SC_ and PCE in this device were slightly lower than those of the reference device, its performance was still superior to that of colorful dye-sensitized solar cells, indicating it could be used for BIPV applications [34–36].

Consistent trends in the photovoltaic performance among 20 different PSC devices were observed. Their photovoltaic parameters are summarized in Table 1. Great reproducibility was also verified by histograms (Fig. S1) and box plots (Fig. S2) of the device performance parameters (*J*_SC_, open circuit voltage (*V*_oc_), fill factor (FF), and PCE) for individually fabricated devices. As can be seen in Table 1 and Fig. S2, changes in optical properties did not influence the photovoltaic parameters of *V*_oc_ and FF. This was also indicated in the plot of photovoltaic enhancement (%) shown in Fig. 4d. The trends in PCE variation were accompanied by similar changes in *J*_SC_. Even hysteresis behavior in the PSCs was not affected by the moth-eye PDMS as PSCs with and without PDMS present the same *J*–*V* curves regardless of the scan direction (Fig. S3).

**Table 1:** Average performance values from 20 PSCs with and without moth-eye PDMS films

Average	*J*_sc_ (mA cm^−2^)	*V*_oc_ (V)	FF	PCE (%)
300 nm pattern	24.53 ± 0.39	1.03 ± 0.02	77.14 ± 1.24	19.44 ± 0.64
1000 nm pattern	21.11 ± 1.01	1.02 ± 0.03	75.47 ± 3.45	16.20 ± 0.82

## Conclusions

In summary, we have fabricated moth-eye inspired functional PDMS films that improve the performance of PSCs using a robust soft lithography method. The nanostructured PDMS films displayed good structural fidelity and attached readily to the transparent substrates without requiring adhesives. We created high-efficiency PSCs with PCEs exceeding 20% by enhancing the LHE with 300-nm periodic structures. We have also constructed colorful photovoltaic devices by applying 1000-nm moth-eye PDMS films. The iridescent color of these devices can be attributed to the diffraction grating effect. We predict the effective utilization of this bioinspired nano-patterning technology to contribute to the advancement of efficient PSCs.

## Electronic Supplementary Material

Below is the link to the electronic supplementary material.

Supplementary material 1 (PDF 286 kb)
