# Correction to: Moth-eye Structured Polydimethylsiloxane Films for High-Efficiency Perovskite Solar Cells

**DOI:** 10.1007/s40820-019-0325-6

**Published:** 2019-11-01

**Authors:** Min-cheol Kim, Segeun Jang, Jiwoo Choi, Seong Min Kang, Mansoo Choi

**Affiliations:** 1 Global Frontier Center for Multiscale Energy Systems, Seoul National University, Seoul, 151-744 Korea; 2 Department of Mechanical and Aerospace Engineering, Seoul National University, Seoul, 151-744 Korea; 3 Department of Mechanical Engineering, Hanbat National University, Daejeon, 34158 Korea; 4 Department of Mechanical Engineering, Chungnam National University, Daejeon, 34134 Korea

## Correction to: Nano-Micro Lett 10.1007/s40820-019-0284-y

In the original publication, the equal contribution information was not available in first page of the article.

The correct information is provided in this correction.

M.-c. Kim, S. Jang contributed equally to this work.

