# Supplementary material for: Moth-eye Structured Polydimethylsiloxane Films for High-Efficiency Perovskite Solar Cells
Source: Nanomicro Lett. 2019 Jun 25;11:53. doi: 10.1007/s40820-019-0284-y (PMC7770823; doi:10.1007/s40820-019-0284-y)
Supplement: Supplementary file 1 — Supplementary material 1 (PDF 286 kb) [file 40820_2019_284_MOESM1_ESM.pdf]

Supporting Information for

Moth-eye Structured Polydimethylsiloxane Films for High-efficiency Perovskite Solar Cells

Min-cheol Kim[1, 2,#], Segeun Jang[3,#], Jiwoo Choi[1, 2], Seong Min Kang[4,*], Mansoo Choi[1, 2,*]

[1]Global Frontier Center for Multiscale Energy Systems, Seoul National University, Seoul 151-744, Korea

[2]Department of Mechanical and Aerospace Engineering, Seoul National University, Seoul 151-744, Korea

[3]Department of Mechanical Engineering, Hanbat National University, Daejeon 34158, Korea

[4]Department of Mechanical Engineering, Chungnam National University, Daejeon 34134, Korea

[#]Min-cheol Kim and Segeun Jang contribute equally to this work

*Corresponding authors. E-mail: smkang@cnu.ac.kr (Seong Min Kang); mchoi@snu.ar.kr (Mansoo Choi)

Supplementary Figures

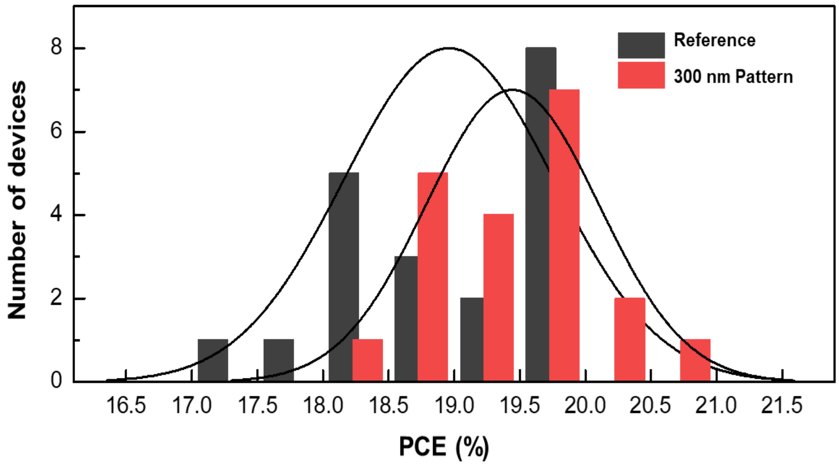

Fig. S1 The PCE distribution histogram of the perovskite solar cell with and without moth-eye PDMS pads for 20 devices

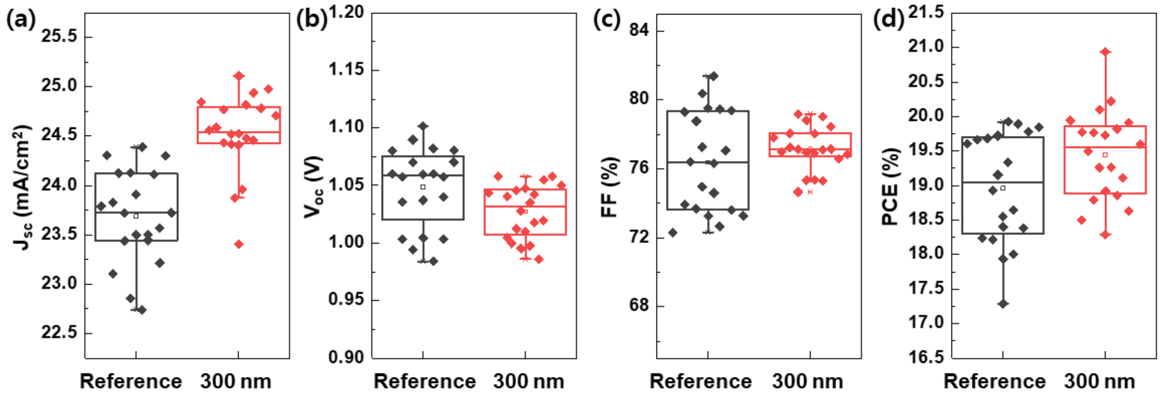

Fig. S2 Photovoltaic parameters (**a** J_{sc}, **b** V_{oc}, **c** FF, **d** PCE) distribution box chart of the perovskite solar cell with and without moth-eye PDMS pads for 20 devices

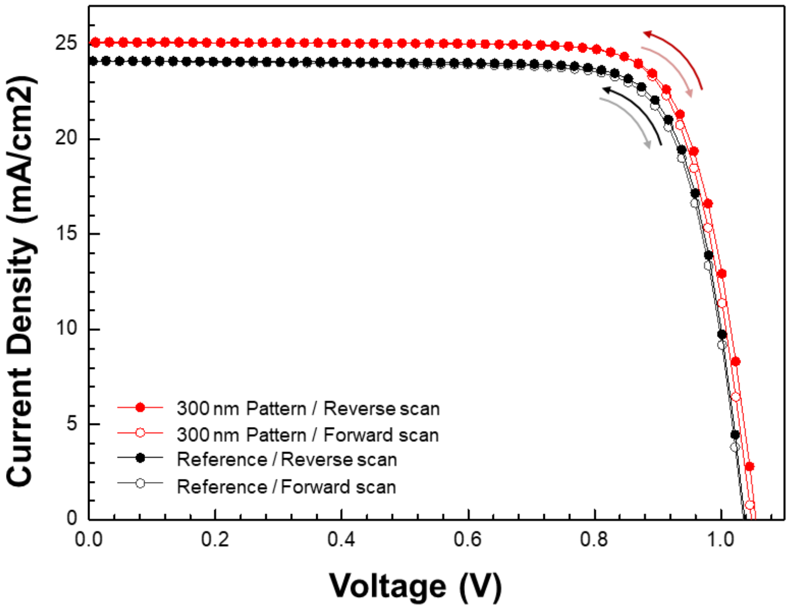

Fig. S3 Comparison of hysteresis behavior for the perovskite solar cell with and without moth-eye PDMS pads by measuring reverse and forward scan of J-V curves
